# Selective mutism due to a dog bite trauma in a 4-year-old girl: a case report

**DOI:** 10.1186/1752-1947-3-100

**Published:** 2009-11-03

**Authors:** Dimitrios Anyfantakis, Emmanouil Botzakis, Evangelos Mplevrakis, Emmanouil K Symvoulakis, Ioannis Arbiros

**Affiliations:** 1 Department of Pediatric Surgery, University General Hospital of Heraklion, Crete, Greece; 2 Department of Child and Adolescent Psychiatry, University General Hospital of Heraklion, Crete, Greece; 3 Department of Blood Donation, University General Hospital of Heraklion, Crete, Greece

## Abstract

**Introduction:**

A child experiencing an event of threatening or catastrophic nature may experience considerable post-traumatic psychological distress. Dog bites present an important public health problem and are a frequent cause of physical trauma in children. Physicians who manage paediatric trauma may not be vigilant of the high risk of psychological stress in children exposed to a physical injury.

**Case presentation:**

A 4-year-old white girl of Greek origin, with a dog-bite related trauma was admitted to the University Hospital of Crete, Greece, for surgical repair and intravenous antibiotic therapy due to extensive lesions. Exposure to the traumatic event triggered the onset of an unusual psychological response, selective mutism and acute post-traumatic stress disorder.

**Conclusion:**

There is limited literature discussing the psychological effect of dog bites in children. Parents and physicians involved in pediatric physical trauma need to be more familiar with post-traumatic behavioral reactions. Awareness of the potential development of such reactions may result in early detection and effective management of children at risk.

## Introduction

Phenomena that involve serious injuries and produce intense fear, helplessness, or horror may result in many symptoms of post-traumatic stress disorder (PTSD) [1]. Injured children seem to be more vulnerable than adults to developing significant psychological distress [2]. Low levels of diagnostic accuracy are partially attributed to the limited awareness among physicians of the potential development of acute or chronic post-traumatic stress reactions after a physical trauma [2].

Dog bites represent a frequent cause of physical trauma among children [3]. However, their psychological impact on paediatric care seems to be underestimated [4]. We report an unusual psychological reaction in a child after a dog attack.

## Case presentation

A 4-year-old white girl from Greece was attacked by a dog owned by her neighbour while playing unsupervised in front of her yard. The child was transported to the emergency department by the dog owner.

On admission, she was confused and lethargic, presenting findings compatible with hypovolemic shock (heart rate 130 beats per minute and hemoglobin level of 7.8 g/dl) secondary to traumatic blood volume loss. Hemodynamic compromise required an aggressive intravenous fluid administration and blood transfusion. Physical examination revealed multiple deep scalp lacerations. After rigorous disinfection, surgical repair was performed in the hospital's operating unit. Due to the extensive nature of the traumatic lesions and the subsequent high risk of infection, the healing process required two weeks of intravenous antibiotic therapy. Rabies prophylaxis was not administered due to the documented rabies vaccination status of the dog.

On the second day of hospitalization, the child was in a depressed mood and displayed mild withdrawal from contact with others. A psychiatric evaluation was performed. During consultation, the child was apparently agitated and refused to participate in any conversation. Non-verbal communication was used instead, including gestures and shaking of the head. The behaviour had not been present before the dog attack. On the sixth day of hospitalization, the child talked for the first time to her mother and asked her: "Where were you when the dog attacked me?".

After a complete suture removal 15 days after the injury, she was discharged. Psychiatric monitoring was arranged after two months. During this interval, the child refused to speak to physicians and other children in the neighbourhood, and used only gestures to communicate while engaging in normal conversation in the home setting. Her memories of the dog attack remained remarkably clear. For six weeks as an outpatient, the child had recurrent traumatic memories when questioned about dogs. After this interval, the girl manifested a persistent avoidance of thoughts and conversations associated with the event. Remarkably, the parents reported that the child was avoiding the dog owner as well as the place where the dog attack occurred. Feelings of estrangement from her neighbours were also present. Hyperarousal occurred in the form of outbursts of anger and anxiety when left alone. She also had difficulty concentrating.

A limited expression of emotions and a reluctance to play with toy dogs were observed during psychiatric consultation. This case fulfilled all diagnostic criteria for selective mutism and PTSD according to the Diagnostic and Statistical Manual of Mental Disorders, (4^th ^edition) [1]. Psychological treatment consisted of supportive psychotherapy for the child and consecutive sessions of counseling for her parents. On her six-month follow-up appointment a symptomatic improvement was evident, with decreased levels of anxiety and normal rates of social and verbal interaction. During consultation, the girl was clearly less anxious and able to communicate her needs verbally. According to her parents, she had become more comfortable speaking in environments out of the home setting and playing with other children in the place where the dog attack occurred.

## Discussion

In this case, the dog attack was associated with an unpredicted psychological morbidity, triggering the onset of selective mutism and acute PTSD. First described by Kussmaul in 1877, selective mutism was named 'aphasia voluntaria', highlighting the voluntary decision not to speak in certain situations [5]. The main diagnostic feature of the disorder is a persistent lack of speech in special social settings where speaking is expected, despite normal speech in other situations [1]. Time of onset is usually before the age of 5 years [5]. Once it starts selective mutism has a variable course, lasting for a few months in some cases or persisting for years in others [5]. Selectively mute children often rely on different types of communication such as gesturing, shaking the head, pulling or pushing [5]. It is a rare clinical entity, found in fewer than 1% of individuals, with a small preponderance in girls [5].

A variety of etiological theories have been suggested for selective mutism [5]. Symptom development has also been reported after a traumatic experience such as sexual abuse [6], divorce and the death of a loved one [7]. Its presence has been associated with impairment of the socialisation and school performance of the child [8]. Although well documented, selective mutism remains a poorly understood and under-recognised disorder in children under school-age [5]. In a primary care survey, the limited familiarity of physicians with the diagnostic features and management of selective mutism resulted in considerable misdiagnosis and delays in the referral process [8]. It is remarkable that in the survey by Schwartz et al., almost 7 out of 10 children with selective mutism never received an accurate diagnosis, and in approximately half of these cases, the reluctance to speak was wrongly attributed to shyness [8]. The ability of the child to speak normally in the home setting with parents is partially responsible for the underestimation of the disorder and the existent lag between onset and time of referral [5]. Current management involves behavioural therapy, family therapy and in some cases pharmacotherapy [5].

PTSD is a highly prevalent condition among children exposed to a life-threatening or distressing event [9]. Dog bites represent an important public health problem, with children under 10 years old being at the highest risk of experiencing injuries to the face, head and neck area [3]. The burden of the problem in terms of the psychological domain is notable, as children exposed to dog attacks experience significant emotional distress and behavioural dysfunction [10]. However, the increased risk of psychological consequences in children after physical trauma is often overlooked [2]. In a study among child victims of dog bites, despite the high occurrence of post-traumatic psychological morbidity, psychological support was not provided [4].

Low rates of diagnosis for post-traumatic psychological disability are partially attributed to parents [9]. It has been reported that they often tend to minimize the post-traumatic emotional response of their children and are reluctant to seek psychological support for their distress [9]. Early detection and prompt initiation of treatment represent the key issues for the management of both PTSD [4] and selective mutism [8]. Treatment requires both psychological and pharmacological interventions.

## Conclusion

The issue of childhood psychological distress after dog bites has not been extensively reported in the literature. This case report highlights the necessity of health professional and parental awareness of post-traumatic psychiatric morbidity in children subsequent to a physical trauma. Having knowledge of these disorders may be helpful in the early detection of children at risk and to coordinate effective counseling, psychological support and follow-up.

## Consent

Written informed consent was obtained from the patient's next-of-kin for publication of this case report and any accompanying images. A copy of the written consent is available for review by the Editor-in-Chief of this journal.

## Competing interests

The authors declare that they have no competing interests.

## Authors' contributions

DA and EB conceived the idea. DA drafted and prepared the manuscript. EB, VM and IA carried out the review of the patient's medical record in order to collect all the available information. EKS and DA collected the follow-up information. EKS provided clinical details and technical input, revised the manuscript and performed editing and format changes throughout the manuscript.
